# Do Serologic Domains of the 2023 ACR/EULAR Classification Criteria for Antiphospholipid Syndrome Define Distinct Clinical Subgroups During Pregnancy?

**DOI:** 10.3390/ijms27146100

**Published:** 2026-07-08

**Authors:** Sara del Barrio-Longarela, José L. Hernández, Ana Merino, Leyre Riancho-Zarrabeitia, Alejandra Comins-Boo, Marcos López-Hoyos, Rafael Gálvez-Sánchez, Víctor M. Martínez-Taboada

**Affiliations:** 1 Division of Obstetrics and Gynecology, Hospital Universitario Marqués de Valdecilla, 39008 Santander, Spain; saradbl11@gmail.com (S.d.B.-L.);; 2 Department of Internal Medicine, Hospital Universitario Marqués de Valdecilla-IDIVAL (Instituto de Investigación Valdecilla), 39008 Santander, Spain; hernandezjluis@gmail.com; 3 Departamento de Medicina y Psiquiatría, Universidad de Cantabria, 39008 Santander, Spain; 4 Rheumatology Department, Hospital Sierrallana-IDIVAL (Instituto de Investigación Valdecilla), 39300 Torrelavega, Spain; 5 Immunology Department, Hospital Universitario Marqués de Valdecilla-IDIVAL (Instituto de Investigación Valdecilla), 39011 Santander, Spain; 6 Departamento de Biología Molecular, Universidad de Cantabria, 39011 Santander, Spain; 7 Division of Rheumatology, Hospital Universitario Marqués de Valdecilla-IDIVAL (Instituto de Investigación Valdecilla), 39008 Santander, Spain

**Keywords:** pregnancy morbidity, antiphospholipid syndrome, antiphospholipid antibodies, classification criteria, ACR/EULAR criteria, autoimmunity, serologic burden

## Abstract

To evaluate the clinical applicability of the 2023 ACR/EULAR serologic classification criteria for antiphospholipid syndrome (APS) in a cohort of pregnant women across the APS spectrum, and to assess the association between serologic burden and clinical manifestations, obstetric outcomes, and treatment response, a retrospective cohort study was conducted including 190 pregnant women with persistent antiphospholipid antibody (aPL) positivity. Patients were classified according to the ACR/EULAR serologic criteria into two groups: high serologic burden (≥3 points) and low serologic burden (≤2 points). Clinical, obstetric, therapeutic, and reproductive variables were compared between groups. Nearly one-third of women did not meet the 2023 ACR/EULAR serologic threshold. Although most patients with thrombotic or obstetric APS fulfilled the ACR/EULAR serologic threshold, 28% of women with obstetric APS and 35.3% of those with pregnancy-related morbidity had ≤2 serologic points. Baseline demographic characteristics were similar between groups. Obesity (23.9% vs. 10%; *p* = 0.04) and obstetric comorbidity (17.9% vs. 5.4%; *p* = 0.02) were more frequent among patients with higher serologic burden. Rates of obstetric complications were comparable between groups. The most commonly prescribed treatment was low-dose aspirin combined with low-molecular-weight heparin, with no differences according to serologic classification. Live birth rates and adverse pregnancy outcomes were comparable between groups, with favorable outcomes observed in treated pregnancies. Serologic burden as defined by the 2023 ACR/EULAR criteria does not reliably predict obstetric complications, nor identify a clinically more severe subgroup. These findings highlight that clinical judgment beyond formal classification frameworks remains essential when managing pregnant women with antiphospholipid antibodies.

## 1. Introduction

Antiphospholipid syndrome (APS) is an autoimmune disorder characterized by thrombotic events and/or pregnancy morbidity in the presence of persistently positive antiphospholipid antibodies (aPLs). Since the publication of the revised Sapporo classification criteria in 2006, APS has been defined using a binary model based on the presence or absence of clinical and laboratory criteria, without weighting the relative clinical relevance of individual manifestations or autoantibody profiles [1]. Recently, the classification of APS has undergone substantial revision, culminating in the proposal of the 2023 American College of Rheumatology/European Alliance of Associations for Rheumatology (ACR/EULAR) classification criteria [2]. These criteria were developed to provide a more specific and standardized framework than the revised Sapporo criteria, introducing a weighted, semiquantitative scoring system that restructures the diagnostic value of the different clinical and serologic domains of APS. One of the main innovations of the 2023 ACR/EULAR criteria is the redefinition of the serologic domains. While previous criteria considered the qualitative presence of one or more aPLs (lupus anticoagulant (LA), anticardiolipin antibodies (aCLs), or anti-β2-glycoprotein I antibodies (anti-β2GPIs)) as equivalent, the new system assigns different weights according to antibody type, isotype, and persistence. Persistent LA positivity receives the highest serologic score, followed by IgG aCLs or anti-β2GPIs, whereas IgM isotypes are assigned a lower value, even at high titers. In addition, except for isolated LA, persistent positivity in at least two determinations separated by 12 weeks is required, reinforcing the importance of serologic stability [2].

This reweighting reflects accumulating evidence over the past two decades demonstrating that not all aPLs confer the same clinical risk. In particular, LA has consistently been identified as the aPL most strongly associated with thrombotic and obstetric manifestations of APS, surpassing isolated aCL or anti-β2GPI positivity [2,3,4,5]. The decision to assign greater weight to LA and combined antibody profiles was based on a rigorous evidence-based methodological process, including systematic literature reviews, international Delphi exercises, and multicriteria decision analysis (MCDA) [2,3]. This approach allowed identification of the clinical and serologic domains most robustly associated with APS in both thrombotic and obstetric settings, and the subsequent adjustment of scoring thresholds to maximize specificity. As a result, the 2023 ACR/EULAR criteria have demonstrated high specificity and improved homogeneity in classified APS populations, particularly in research settings [5,6,7]. However, increasing evidence suggests that this gain in specificity may come at the expense of reduced sensitivity, especially in patients with predominantly obstetric manifestations [8,9,10,11].

The obstetric domain represents a particularly critical aspect of the new classification system. Unlike the revised Sapporo criteria, which assigned distinct diagnostic weight to recurrent early pregnancy loss (<10 weeks) and single fetal death beyond 10 weeks’ gestation, the 2023 ACR/EULAR criteria equate early pregnancy losses and fetal deaths between 10 and 16 weeks, assigning them the lowest possible obstetric score. Moreover, the obstetric items that receive the highest weight, such as severe preeclampsia or placental insufficiency before 34 weeks’ gestation, are relatively infrequent in routine clinical practice. As a consequence, common obstetric manifestations of APS, including recurrent early pregnancy loss, may be underrepresented in the final classification score [2,11]. Several validation studies have consistently reported that a substantial proportion of women with clinically evident obstetric APS do not reach the required threshold for classification under the new criteria, despite fulfilling the revised Sapporo criteria [6,7,8,9,10,11]. This loss of sensitivity appears to disproportionately affect patients with exclusively obstetric manifestations and those with lower serologic burden, particularly isolated or predominant IgM positivity, which is common in obstetric APS [8,9,10,11].

Furthermore, most published validation cohorts include mixed populations with primary and secondary APS or patients with associated systemic autoimmune diseases. This may lead to an overrepresentation of infrequent clinical domains, such as microvascular disease or thrombocytopenia, while limiting the generalizability of findings to women with primary obstetric APS. Data focusing specifically on European cohorts of women with primary obstetric APS and no associated systemic autoimmune disease remain scarce. In this context, recent studies, including our own, have suggested that although the 2023 ACR/EULAR criteria represent an important advance in APS classification, their strict application may limit the identification and appropriate treatment of women with obstetric APS [10,11]. These findings highlight the need to critically assess the clinical applicability of the new criteria, particularly in obstetric settings, and to reinforce the role of clinical judgment beyond classification frameworks. Moreover, the applicability of APS classification criteria to multiple pregnancies warrants consideration, as these pregnancies carry an inherently increased risk of obstetric complications and remain underrepresented in available validation studies.

Therefore, the present study aims to evaluate the clinical applicability of the serologic domains of the 2023 ACR/EULAR classification criteria in a monocentric cohort of women with aPL positivity during pregnancy. By comparing patients with high (≥3 points) and low (≤2 points) serologic burden, we sought to determine whether serologic stratification correlates with clinical phenotype, obstetric outcomes, and therapeutic benefit, and to explore the diagnostic and prognostic implications of these findings in routine clinical practice.

## 2. Results

### 2.1. Serologic Classification of the Study Cohort

A total of 190 pregnant women with persistent aPL positivity according to the revised Sydney classification criteria for APS were included [1]. Patients were classified according to the 2023 ACR/EULAR serologic criteria into two groups: those who fulfilled the new criteria [high serologic burden (≥3 points)] and those who did not meet the criteria [low serologic burden (≤2 points)] [2]. Interestingly, 56 (29.47%) women did not fulfill the new serologic criteria for APS.

**Figure 1:**
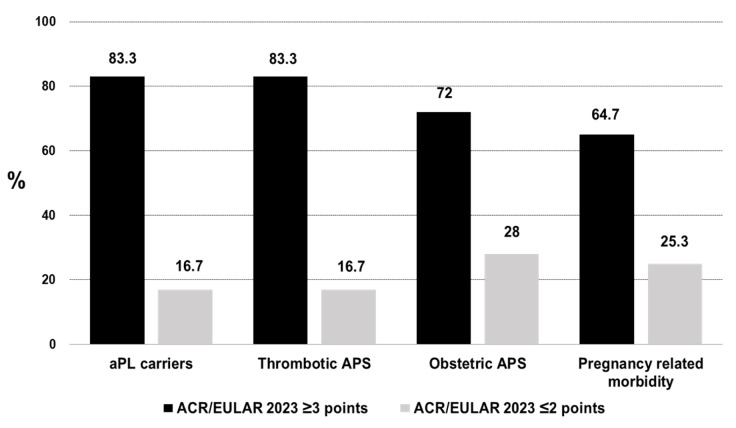
Study groups of patients who fulfilled ACR/EULAR 2023 serologic classification criteria. ACR/EULAR 2023 ≥ 3 points: D7 and/or D8. ACR/EULAR 2023 ≤ 2 points: D7 (Lupus anticoagulant single) and/or D8 (A). Domain 7: Lupus anticoagulant. Domain 8: Anticardiolipin antibodies/antiβglicoprotein I antibodies: (A) Moderate or high IgM + (aCL and/or AB2GPI); (B) Moderate IgG + (aCL+ and/or AB2GPI); (C) High IgG + (aCL+ or AB2GPI); (D) High IgG + (aCL+ and AB2GPI).

When patients were stratified according to clinical presentation, most asymptomatic aPL carriers (83.3%), patients with thrombotic APS (83.3%), and those with obstetric APS (72%) fulfilled the serologic threshold of ≥3 points. However, 28% of patients with obstetric APS and 35.3% of those with pregnancy-related morbidity had ≤2 serologic points and therefore would not meet the ACR/EULAR 2023 classification threshold (Figure 1 and Supplementary Table S1).

### 2.2. Baseline Demographic Characteristics, Cardiovascular Risk Factors, and Comorbidities

Baseline characteristics according to serologic burden are summarized in Table 1. Mean age was similar between patients with high and low serologic burden (33.7 ± 5.5 vs. 35.1 ± 6.1 years; *p* = 0.11). Time to diagnosis tended to be longer in patients with ≥3 points (median 22 months [IQR 10–46] vs. 12.5 months [IQR 6.75–32.25]), although this difference did not reach statistical significance (*p* = 0.07). Follow-up duration after diagnosis was comparable between groups (*p* = 0.60).

The overall prevalence of cardiovascular risk factors was similar in both groups (*p* = 0.97). However, obesity was significantly more frequent among patients with ≥3 points (23.9% vs. 10%; *p* = 0.04), whereas no significant differences were observed in smoking status (*p* = 0.44), hypertension (9% vs. 1.8%; *p* = 0.11), diabetes mellitus (*p* = 0.56), or dyslipidemia (*p* = 0.53).

Regarding comorbidities, obstetric comorbidity was significantly more frequent in patients with high serologic burden (17.9% vs. 5.4%; *p* = 0.02). In contrast, inherited thrombophilia (*p* = 0.26) and thyroid disease (*p* = 0.28) did not differ significantly between groups.

### 2.3. Distribution of Clinical APS Domains According to the 2023 ACR/EULAR Criteria

The distribution of clinical domains according to the 2023 ACR/EULAR classification criteria is shown in Table 2. Macrovascular thrombotic manifestations were infrequent and similarly distributed between groups. Venous thromboembolism occurred in 7.5% of patients with ≥3 points and 3.6% of those with ≤2 points (*p* = 0.51), while arterial thrombosis was observed in 3.7% and 1.8%, respectively (*p* = 0.67). No cases of microvascular involvement were identified.

The obstetric domain was present in similar proportions in both groups (38.8% vs. 39.3%; *p* = 0.95). Recurrent early pregnancy loss and/or fetal death before 16 weeks occurred at comparable rates (59.6% vs. 59.1%). Fetal death between 16 and 33 weeks in the absence of preeclampsia or placental insufficiency was slightly more frequent in patients with ≤2 points (31.8% vs. 25%), while severe preeclampsia or severe placental insufficiency before 34 weeks occurred at similar frequencies (11.5% vs. 9.1%). The coexistence of severe preeclampsia and placental insufficiency was observed only in patients with ≥3 points (3.8%).

Thrombocytopenia tended to be more frequent in patients with high serologic burden (14.2% vs. 7.1%), although this difference did not reach statistical significance (*p* = 0.17).

### 2.4. Sydney Criteria and Obstetric Morbidity According to Serologic Burden

Clinical manifestations included in the revised Sydney criteria and non-criteria obstetric morbidity are detailed in Table 3 and Supplementary Table S4.

The median number of pregnancies was similar in both groups (3 [IQR 2–4] vs. 3 [IQR 2–4]; *p* = 0.35). Early recurrent pregnancy loss (<10 weeks) occurred at similar rates (*p* = 0.72). Fetal death beyond 10 weeks (*p* = 0.23), preterm birth before 34 weeks (*p* = 0.73), and thrombotic events (*p* = 0.21) were more frequent in patients with ≥3 points, although these differences did not reach statistical significance. As previously known, confirmed LA and IgG subtypes were associated with poor obstetric morbidity [12,13].

Regarding non-criteria obstetric morbidity, preterm delivery between 34 and 37 weeks (*p* = 0.79) and late-onset preeclampsia/eclampsia (>34 weeks) (*p* = 0.99) occurred at similar rates. Abruptio placentae was observed only in patients with ≤2 points (3.6%), showing a trend toward significance (*p* = 0.08). Repeated in vitro fertilization failures (>2 cycles) were more frequent in patients with ≤2 points (17.9% vs. 9.7%; *p* = 0.12), while early pregnancy loss (<10 weeks) tended to be more frequent in patients with ≥3 points (44.8% vs. 33.9%; *p* = 0.17).

### 2.5. Treatment Patterns According to Serologic Classification

**Figure 2:**
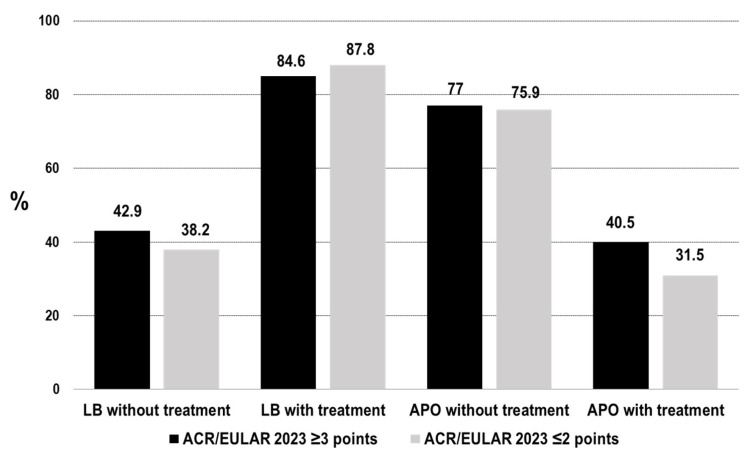
Main adverse pregnancy outcomes (APO) and live birth (LB) rates according to the ACR/EULAR 2023 serologic classification criteria. LB: live birth; APO: adverse pregnancy outcomes.

The main treatment strategies are summarized in Figure 2 and Supplementary Table S2. The combination of low-dose aspirin (LDA) and low-molecular-weight heparin (LMWH) was the most frequently prescribed regimen in both groups (61.9% vs. 53.6%; *p* = 0.28). LDA monotherapy was used in 29.1% of patients with ≥3 points and 33.9% of those with ≤2 points (*p* = 0.51). Corticosteroids (*p* = 0.73) and antimalarial agents (*p* = 0.40) were infrequently prescribed, with no significant differences between groups.

### 2.6. Reproductive Outcomes and Adverse Pregnancy Outcomes

**Figure 3:**
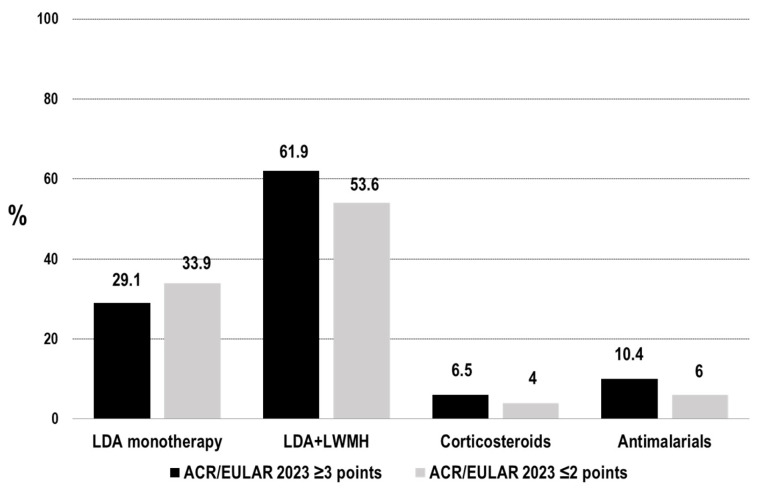
Main treatments in the different study groups according to the serologic burden. LDA: low-dose aspirin; LMWH: low-molecular-weight heparin.

Reproductive outcomes according to serologic classification are shown in Figure 3 and Supplementary Table S3. Total live birth rates were high and comparable between patients with ≥3 and ≤2 points (97.6% vs. 100%; *p* = 0.57). Among untreated pregnancies, live birth rates were similar between groups (*p* = 0.56), while treated pregnancies showed markedly higher live birth rates in both groups (*p* = 0.62).

The overall rate of APO did not differ significantly between groups (82.1% vs. 80.4%; *p* = 0.78). Treatment was associated with a reduction in APO in both serologic groups (40.5% vs. 31.5%; *p* = 0.25).

## 3. Discussion

The present study demonstrates that a substantial proportion of pregnant women within the spectrum of APS do not meet the serologic threshold established by the 2023 ACR/EULAR classification criteria. This finding raises concerns regarding potential under-classification of obstetric APS and is consistent with recent validation studies highlighting the need for comprehensive clinical assessment beyond strict serologic scoring [5,7,8,9,11,14,15,16].

Notably, our data reveal that the serologic subtypes defined by the 2023 ACR/EULAR criteria are not associated with a worse clinical prognosis in this cohort. Neither the incidence of obstetric events nor the severity of pregnancy complications differed significantly between patients with low (≤2 points) and high (≥3 points) serologic burden. These findings indicate that the cumulative serologic score does not reliably discriminate a clinically more severe subgroup within the obstetric APS spectrum. This lack of association was consistent across all clinical domains evaluated. Patients with ≤2 serologic points, who would remain unclassified under the new framework, experienced obstetric complications of similar frequency and severity to those observed in patients fulfilling the classification threshold. This observation is particularly relevant for the obstetric domain, where severe manifestations such as fetal death, preeclampsia with severe features, or placental insufficiency were also documented in patients with low serologic scores. These data reinforce concerns that the current weighting of obstetric items in the 2023 ACR/EULAR criteria may inadequately capture the full clinical spectrum of obstetric APS [10,17]. Our findings align with those reported by Zhao et al. [5], who observed that a significant proportion of patients with a typical APS phenotype failed to meet the ACR/EULAR classification criteria due to insufficient serologic weighting. Similarly, Foddai et al. [7] and Tang et al. [6] have emphasized that the increased specificity of the 2023 framework is accompanied by a concomitant loss of sensitivity, potentially excluding clinically relevant patients without providing a clear clinical benefit. This is particularly evident in cases of recurrent pregnancy loss (RPL). In agreement with the strictness of the new criteria [2], a recent study of 165 patients with unexplained RPL found that the 2023 ACR/EULAR criteria reclassified nearly all cases (98.8%) as idiopathic, identifying a prevalence of only 1.2% compared to 14.5% under the Sydney criteria [18]. Such rarity suggests that the clinical impact of serologic screening in RPL requires further validation through large-scale collaborative trials to refine treatment strategies.

Furthermore, the potential limitations of these criteria become apparent when examining treatment patterns. In our study, therapeutic strategies were driven predominantly by clinical history, such as prior pregnancy loss or thrombosis, rather than the serologic score itself, reflecting real-world clinical practice. More importantly, treatment was associated with a marked improvement in reproductive outcomes regardless of serologic burden. Live birth rates increased substantially, and APO were reduced by approximately half in both groups. This demonstrates that women with a low serologic burden derive a clear clinical benefit from prophylactic treatment, despite their exclusion from formal classification. These results reinforce our previous work, in which we questioned the suitability of the 2023 ACR/EULAR criteria for advancing obstetric APS research, noting that while they improve cohort homogeneity, they may overlook patients with incomplete or atypical serologic profiles [10,11]. Taken together, our findings support the growing consensus that the 2023 ACR/EULAR criteria should be regarded primarily as a research classification tool rather than a diagnostic or prognostic instrument [7,11,14,16,17]. In clinical practice, individualized management remains essential, and clinical judgment informed by obstetric history and overall risk assessment should remain central to ensuring that patients with clinically significant disease are not deprived of effective preventive strategies.

The different serologic profiles incorporated into the 2023 ACR/EULAR criteria may reflect distinct immunopathogenic pathways within the APS spectrum. Persistent lupus anticoagulant positivity, high-titer anticardiolipin antibodies, and anti-β2-glycoprotein I antibodies have been associated with different mechanisms of endothelial activation, complement engagement, and thrombo-inflammatory responses. However, in the present cohort, these serologic differences did not translate into clearly distinguishable clinical phenotypes or obstetric outcomes. This observation suggests that serologic burden alone may be insufficient to capture the complexity of disease expression during pregnancy and raises questions regarding the clinical utility of risk stratification based exclusively on conventional antiphospholipid antibody profiles.

An additional consideration is the potential contribution of non-criteria aPLs, which were not assessed in the present study. Increasing evidence suggests that selected non-criteria aPLs may identify patients with clinically relevant APS manifestations despite negative or low-burden conventional serology. In particular, Truglia et al. [19,20] demonstrated that antibodies directed against phosphatidylserine/prothrombin, vimentin/cardiolipin, and carbamylated β2-glycoprotein I are frequently detected in patients with seronegative APS and are associated with both thrombotic and obstetric manifestations. These findings may be especially relevant to our cohort, as women with ≤2 serologic points experienced obstetric complications and appeared to benefit from treatment similarly to those meeting the current classification threshold. It is therefore possible that some patients categorized as having a low serologic burden according to the 2023 ACR/EULAR criteria may harbor additional pathogenic autoantibodies not captured by the current laboratory framework. Whether these antibodies provide incremental predictive value beyond current criteria-based serology remains to be established.

These findings support the need for a more comprehensive approach to patient stratification in obstetric APS. Future studies should integrate conventional serologic markers with emerging biomarkers of immune dysregulation, including complement activation pathways, inflammatory mediators, endothelial dysfunction markers, and molecular signatures associated with placental injury. Such multidimensional approaches may provide a more accurate representation of disease heterogeneity and improve risk prediction beyond current classification frameworks.

Despite the marked similarities observed between pregnant women carrying aPLs from both serologic groups, there are only two differences that stand out and deserve further comment. Notably, patients with a higher aPL burden have a higher frequency of obesity and obstetric comorbidities. Obesity induces a chronic low-grade inflammatory state that alters innate and adaptive immunity, facilitating the loss of immunological tolerance [21]. This proinflammatory environment, mediated by adipokines and cytokines that activate T and B lymphocytes, promotes autoantibody production [22,23,24,25,26]. Furthermore, obesity activates the complement system and triggers endothelial dysfunction, potentially increasing the exposure of phospholipid antigens and perpetuating aPL synthesis [24,25]. While the direct relationship between BMI and aPL positivity remains debated in recent observational studies [27], mechanistic evidence suggests that metabolic inflammation enhances the thrombo-inflammatory mechanisms of aPLs, particularly in obstetric contexts. Evidence also links polycystic ovary syndrome (PCOS) and endometriosis with aPLs, though findings are heterogeneous. A higher prevalence of autoantibodies (aPLs, anti-histone, anti-dsDNA) has been documented in PCOS, but the clinical relevance and direct relationship with aPLs remain controversial [28,29]. In endometriosis, an increased frequency of aPLs has been observed in serum and peritoneal fluid within infertility cohorts, suggesting abnormal immune activation [30,31,32]. However, recent high-throughput studies have not confirmed these differences universally, indicating that the relationship may be subgroup-dependent [33]. Taken together, these results suggest that factors other than autoimmunity, such as metabolic or inflammatory factors, may influence the autoantibody profile and burden in patients with APS. These aspects, particularly in obstetric APS, warrant consideration in future studies.

This study has limitations inherent to its retrospective design. Furthermore, as the research was conducted at a single tertiary center within a specialized multidisciplinary unit for autoimmune obstetric complications, the external validity of our findings may be constrained. Another significant methodological consideration is that the impact of serologic alterations on APOs was analyzed on a per-patient basis rather than per pregnancy. Finally, the relatively limited follow-up of patients, coupled with the dynamic nature of the disease during future pregnancies, could cause some patients to switch from one study group to another.

Conversely, a primary strength of this research lies in its stringent exclusion criteria, which eliminated confounding systemic autoimmune diseases to ensure a more homogeneous study population within the clinical spectrum of APS. Second, our cohort encompasses the entire clinical continuum of suspected APS, ranging from asymptomatic aPL carriers to patients meeting the formal 2006 classification criteria [1]. Moreover, this study also incorporates a clinically significant subgroup of patients with non-criteria obstetric manifestations, which are highly prevalent in routine practice but often overlooked in formal research. Furthermore, the robustness of our data is reinforced by a comprehensive evaluation of serologic profiles, cardiovascular risk factors, and additional comorbidities [34,35,36] that may influence the overall obstetric prognosis.

In summary, while the 2023 ACR/EULAR criteria improve diagnostic specificity, they may reduce sensitivity, particularly in obstetric APS, where nearly one-third of the patients lose the serologic criteria. Serologic burden as defined by these criteria does not reliably predict obstetric complications, nor identify a clinically more severe subgroup. Furthermore, our results suggest that factors other than autoimmunity, such as metabolic or inflammatory ones, may influence the autoantibody profile and burden in APS patients. In pregnant women with aPL positivity, comprehensive assessment beyond formal classification frameworks remains essential to optimize maternal–fetal outcomes.

## 4. Methods and Materials

### 4.1. Study Design and Participants

This retrospective cohort study included 190 pregnant women with persistently positive aPLs within the clinical spectrum of APS, followed at the Autoimmune Diseases Pregnancy Clinic, a multidisciplinary unit of a tertiary care teaching hospital, between January 2005 and December 2024. All patients fulfilled serologic criteria of the revised Sydney classification criteria for APS [1]. Women who fulfilled the classification criteria for rheumatic autoimmune diseases other than APS were excluded. Persistent aPL positivity was defined as positivity in at least two determinations separated by a minimum of 12 weeks, in accordance with international recommendations [1]. The information collected from individual cases was completely anonymized, and the study was approved by the Ethics Committee of Cantabria (internal code: 2025.050). The study was conducted in accordance with the ethical principles of the Declaration of Helsinki.

Patients were classified according to the serologic domains of the 2023 ACR/EULAR classification criteria into two groups: high serologic burden (≥3 points) and low serologic burden (≤2 points). Serologic burden was defined as the cumulative serologic score derived from domains 7 and 8 of the 2023 ACR/EULAR criteria [2]. The study groups are shown in Figure 1 and Supplementary Table S1.

### 4.2. Data Collection

Data were collected retrospectively using a prespecified standardized questionnaire and recorded in a computerized database. We assessed the following clinical variables, including demographic and general characteristics: age, sex, body mass index (BMI), current/past tobacco use, high blood pressure (equal or greater than 140/90 mm Hg or being on antihypertensive agents), dyslipidemia (serum total cholesterol or triglyceride levels greater than 230 mg/dL and 150 mg/dL respectively or being on lipid-lowering drugs), diabetes mellitus (according to the ADA criteria) [37], and past or present family (<50 years) or personal history of thrombotic disease. *Comorbidities* potentially associated with pregnancy outcomes were also recorded, including inherited thrombophilia (factor V Leiden, prothrombin mutation, protein S and/or protein C deficiency), thyroid disease (history of hypo/hyperthyroidism or the presence of confirmed specific autoantibodies), and obstetric comorbidity (local uterine abnormalities, endometriosis, and polycystic ovary syndrome).

### 4.3. Autoantibody Assessment

Anticardiolipin antibodies (aCLs) and anti-beta2 glycoprotein I antibodies (AB2GPI) of the IgG and IgM isotypes were quantified by commercial enzyme immunoassay in solid phase (ELISA; Orgentec Diagnostika GmbH, Mainz, Germany). The results are reported as quantitative and semiquantitative values. Thus, aCLs are quantified in GPL (aCL IgG) or MPL (aCL IgM) according to the standard curve constructed in each test with 5 dilution points of the Harris/Sapporo standards. AB2GPI are quantified as U/mL. Only medium-high titers of aPLs were considered positive [1,2,38]. The criteria recommended by the International Society of Thrombosis and Hemostasis (ISTH) Scientific and Standardization Committee (ISTH) for the standardization of lupus anticoagulant/antiphospholipid antibodies (LA/APA) were applied for the characterization of LA [39,40,41]. Patients with inconclusive serology, defined as persistent low-titer aCLs or AB2GPI and/or intermittent AL, aCLs, or AB2GPI, were excluded from the study.

### 4.4. Clinical Domains

Clinical manifestations were collected according to both the revised Sydney classification criteria [1] and the 2023 ACR/EULAR definitions [2]. Clinical manifestations according to the Sydney criteria in this cohort have been previously reported [42,43,44].

The following ACR/EULAR clinical domains were retrospectively recorded using the strict proposed definitions: macrovascular venous thrombosis (domain 1), macrovascular arterial thrombosis (domain 2), microvascular disease (domain 3), obstetric manifestations (domain 4), cardiac valve disease (domain 5), and thrombocytopenia (domain 6) [2]. These clinical domains are shown in Table 1.

### 4.5. Pregnancy Morbidity Definitions

* *Obstetric manifestations:* (a) Sydney criteria [1]; (b) Non-criteria obstetric morbidity related to APS: 1–2 early pregnancy losses (<10 weeks), preterm birth (between 34 and 36 + 6 weeks), late preeclampsia (>34 weeks), abruptio placentae, and unexplained in vitro fertilization failures (>2) [43,45].
* *Pregnancy loss:* early pregnancy loss (<10 weeks) and/or fetal death (>10 weeks).
* *Adverse pregnancy outcome (APO):* early pregnancy loss, fetal death, preeclampsia, abruptio placentae, and preterm birth (<37 weeks of gestation).

### 4.6. Treatment and Pregnancy Outcomes

Patients were treated according to current guidelines [12,13,46,47]. Information regarding treatment during pregnancy was collected, including low-dose aspirin (LDA) monotherapy, combination therapy with LDA and low-molecular-weight heparin (LMWH), use of corticosteroids, and antimalarial agents. Perinatal outcomes, including live birth and APO, were recorded for all pregnancies and analyzed by serologic groups.

### 4.7. Statistical Analysis

Results were expressed as numbers (percentage), mean ± standard deviation (SD) or median and interquartile range (IQR), as appropriate. Student’s *t*-test, Mann–Whitney U-test, and one-way ANOVA were used to compare quantitative variables, and the Chi-squared and Fisher tests were used to compare categorical data. A two-tailed *p*-value < 0.05 was considered statistically significant in all the calculations. Statistical analyses were conducted using SPSS version 29.0 (IBM Corp., Armonk, NY, USA).

## Figures and Tables

**Table 1:** Demographic characteristics, cardiovascular risk factors, and main comorbidities of patients who fulfilled ACR/EULAR 2023 serologic classification criteria.

	ACR/EULAR 2023 ≥3 Points N = 134	ACR/EULAR 2023 ≤2 Points N = 56	*p*
Age, yrs ± SD	33.7 ± 5.5	35.1 ± 6.1	0.11
Time to diagnosis (m), median [IQR]	22 [10–46]	12.5 [6.75–32.25]	0.07
Follow-up (m), median [IQR]	46 [17–110]	54 [10.25–128]	0.60
**Cardiovascular risk factors, N (%)**	77 (57.5)	32 (57.1)	0.97
- Obesity	28 (23.9)	5 (10)	**0.04**
- Smoking	56 (41.8)	20 (35.7)	0.44
- High blood pressure	12 (9)	1 (1.8)	0.11
- Diabetes	3 (2.2)	0	0.56
- Dyslipidemia	8 (6)	5 (8.9)	0.53
**Comorbidities, N (%)**			
- Hereditary thrombophilia	14 (10.4)	3 (5.4)	0.26
- Thyroid disease	14 (10.4)	9 (16.1)	0.28
- Obstetric comorbidity	24 (17.9)	3 (5.4)	**0.02**

SD: standard deviation; m: months; IQR: interquartile range.

**Table 2:** Clinical APS domains according to the ACR/EULAR 2023 classification criteria.

	ACR/EULAR 2023 ≥3 Points N = 134	ACR/EULAR 2023 ≤2 Points N = 56	*p*
**D1: Macrovascular (VTE), N (%)**	10 (7.5)	2 (3.6)	0.51
**D2: Macrovascular (AT), N (%)**	5 (3.7)	1 (1.8)	0.67
**D3: Microvascular, N (%)**	0	0	
**D4: Obstetric, N (%)**	52 (38.8)	22 (39.3)	0.95
- ≥3 pre-fetal abortion (<10 weeks) and/or fetal (10 w 0 d–15 w 6 d) deaths	31 (59.6)	13 (59.1)	
- Fetal (16 w 0 d–33 w 6 d) deaths in the absence of PEC or IP	13 (25)	7 (31.8)	
- PEC with severe features (<34 w 0 d) or PI with severe features (<34 w 0 d) with/without fetal death	6 (11.5)	2 (9.1)	
- PEC with severe features (<34 w 0 d) and PI with severe features (<34 w 0 d) with/without fetal death	2 (3.8)	0	
**D5: Cardiac valve, N (%)**	0	0	
**D6: Thrombocytopenia, N (%)**	19 (14.2)	4 (7.1)	0.17

D: domain; VTE: venous thrombosis; AT: arterial thrombosis; w: weeks; d: days; PEC: preeclampsia; PI: placenta insufficiency.

**Table 3:** Clinical APS Sydney criteria and obstetric morbidity in patients according to the ACR/EULAR 2023 serologic criteria.

	ACR/EULAR 2023 ≥3 Points N = 134	ACR/EULAR 2023 ≤2 Points N = 56	*p*
Number of pregnancies, median [IQR]	3 [2–4]	3 [2–4]	0.35
**Sydney Criteria, %**			
- Abortion < 10 weeks (≥3)	28 (20.9)	13 (23.2)	0.72
- Fetal death > 10 weeks	27 (20.1)	7 (12.7)	0.23
- Preterm < 34 weeks	8 (6)	2 (3.6)	0.73
- Thrombosis	15 (11.2)	3 (5.4)	0.21
**Obstetric morbidity, %**			
- Abortion < 10 weeks (≤2)	60 (44.8)	19 (33.9)	0.17
- Preterm 34–37 weeks	15 (11.2)	7 (12.5)	0.79
- Preeclampsia/Eclampsia > 34 w	12 (9)	5 (8.9)	0.99
- Abruptio Placentae	0	2 (3.6)	0.08
- IVF failures (>2)	13 (9.7)	10 (17.9)	0.12

IQR: interquartile range; IVF: in vitro fertilization.

## Data Availability

The data underlying this article cannot be publicly shared because they contain sensitive clinical information and are part of an ongoing research project. De-identified data may be made available from the corresponding author upon reasonable request and subject to approval by the Ethics Committee of Cantabria.

## Supplementary Materials

The following supporting information can be downloaded at: https://www.mdpi.com/article/10.3390/ijms27146100/s1.
