# Supplementary material for: Do Serologic Domains of the 2023 ACR/EULAR Classification Criteria for Antiphospholipid Syndrome Define Distinct Clinical Subgroups During Pregnancy?
Source: Int J Mol Sci. 2026 Jul 8;27(14):6100. doi: 10.3390/ijms27146100 (PMC13412096; doi:10.3390/ijms27146100)
Supplement: Supplementary file 1 [file ijms-27-06100-s001.zip › ijms-4391020-supplementary.pdf]

Suppl Table S1.-Study groups in patients who fulfilled ACR/EULAR 2023 serologic classification criteria.

	ACR/EULAR 2023 ≥3 points N=134	ACR/EULAR 2023 ≤2 points N=56
**Asymptomatic aPL carriers, N=12 (%)**	10 (83.3)	2 (16.7)
**Thrombotic APS, N=18 (%)**	15 (83.3)	3 (16.7)
**Obstetric APS, N=75 (%)**	54 (72)	21 (28)
**Pregnancy related morbidity, N=85 (%)**	55 (64.7)	30 (25.3)

ACR/EULAR 2023 ≥3 points: D7 and/or D8.

ACR/EULAR 2023 ≤2 points: D7 (Lupus anticoagulant single) and/or D8 (A).

Domain 7: Lupus anticoagulant.

Domain 8: anticardiolipin antibodies / antib2glicoprotein I antibodies: A) Moderate or high IgM + (aCL and/or AB2GPI); B) Moderate IgG + (aCL+ and/or AB2GPI); C) High IgG + (aCL+ or AB2GPI); D) High IgG + (aCL+ and AB2GPI).

Suppl Table S2.- Main treatments in the different study groups according to the serological burden.

	ACR/EULAR 2023<br>≥3 points<br>N=134	ACR/EULAR 2023<br>≤2 points<br>N=56	*p*
- LDA monotherapy	39 (29.1)	19 (33.9)	0.51
- LDA+LWMH	83 (61.9)	30 (53.6)	0.28
- Corticosteroids	8 (6.5)	2 (4)	0.73
- Antimalarials	13 (10.4)	3 (5.9)	0.40

LDA: low-dose aspirin; LMWH: low-molecular-weight heparin

Suppl Table S3.- Main adverse pregnancy otcomes (APO) and live birth (LB) according to the ACR/EULAR 2023 serologic classification criteria

	**ACR/EULAR 2023<br>≥3 points<br>N=134**	**ACR/EULAR 2023<br>≤2 points<br>N=56**	***p***
**Total live birth**	120 (97.6)	44 (100)	0.57
LB without treatment	51 (42.9)	21 (38.2)	0.56
LB with treatment	99 (84.6)	36 (87.8)	0.62
**Total APO**	110 (82.1)	45 (80.4)	0.78
APO without treatment	97 (77)	41 (75.9)	0.88
APO with treatment	53 (40.5)	17 (31.5)	0.25

LB: live birth; APO: adverse pregnancy outcomes.

Suppl Table S4.- Main adverse pregnancy outcomes according to the antiphospholipid antibody subtypes

	**≥3 pregnancy loss (<10 weeks)**	**≥1 pregnancy loss (>10 weeks)**	**PE, E, or PI (<34 weeks)**
**Lupus anticoagulant, Nº (%)**			
LA isolated	2 (4.9)	2 (5.9)	2 (20)
LA confirmed	9 (21.9)	16 (47)	3 (30)
**Anticardiolipin antibodies Nº (%)**			
IgM	16 (39)	14 (41.2)	5 (50)
IgG	13 (31.7)	14 (41.2)	7 (70)
**Anti-β2-glycoprotein I antibodies Nº (%)**			
IgM	16 (39)	11 (32.4)	4 (40)
IgG	11 (26.8)	11 (32.4)	6 (60)

LA. Lupus anticoagulant; PE: preeclampsia; E: eclampsia; PI: placenta insufficiency
